# Effectiveness of Robotics in Stroke Rehabilitation to Accelerate Upper Extremity Function: Systematic Review

**DOI:** 10.1155/2023/7991765

**Published:** 2023-10-27

**Authors:** Cora Carrillo, Devyn Tilley, Kaitlyn Horn, Michelle Gonzalez, Cassidy Coffman, Claudia Hilton, Karthik Mani

**Affiliations:** University of Texas Medical Branch, Galveston, Texas, USA

## Abstract

**Objective:**

To examine the effectiveness of robot-assisted therapy (RAT) combined with conventional therapy (CT) compared to CT alone in accelerating upper extremity (UE) recovery poststroke. *Data Sources*. We searched five databases: Ovid, MEDLINE, CINAHL, PubMed, and Scopus Study Selection. Studies were selected for this review using the following inclusion criteria: randomized controlled trials of adults, RAT combined with CT compared to CT, and Fugl-Meyer Assessment (FMA) as an outcome measure. Studies focused on children with neurological impairments, and studies that used RAT to facilitate lower extremity recovery and/or improve gait were excluded. *Data Extraction*. The initial search yielded 3,019 citations of articles published between January 2011 and May 2021. Fourteen articles met the inclusion criteria. Randomization, allocation sequence concealment, blinding, and other biases were assessed. *Data Synthesis*. Current evidence suggests that the use of RAT along with CT may accelerate upper extremity recovery, measured by FMA, in the beginning of rehabilitation. However, the progress fades over time. More empirical research is needed to validate this observation. Also, the findings related to cost-benefit analyses of RAT are inconclusive.

**Conclusions:**

It is unclear whether RAT accelerates UE recovery poststroke when used in conjunction with conventional therapy. Given the capital and maintenance costs involved in developing and delivering RAT, more controlled studies examining the effectiveness and cost-benefit analysis of RAT are needed before it can be used widely. This trial is registered with CRD42021270824.

## 1. Introduction

Technological gains in rehabilitation have not only impacted the way that diagnostic testing is done, made way for less invasive surgical procedures, and allowed for the online delivery of telehealth services but have also impacted the way that direct rehabilitation can be delivered [1, 2].

The development of new technology, including robots, has been a driving force for improving the treatment for various impairments. According to Chang and Kim, a robot is “a re-programmable, multi-functional manipulator designed to move material, parts, or specialized devices through variable programmed motions to accomplish a task” [3]. The first robotic device designed for rehabilitation was created in 1992 and paved the way for robot-assisted therapy (RAT) [4]. Since then, RAT has been used to treat a wide range of skill deficits or functional impairments such as impaired social and intellectual skills, sensorimotor deficits, gait dysfunctions, decreased hand function, and decreased activity participation in clients with cerebral palsy, autism, spinal cord injury, ankle injuries, intellectual disabilities, and stroke [5–14]. RAT allows for repetitive practice in conjunction with conventional therapies.

Every year, more than 795,000 people have a cerebrovascular accident (stroke) in the USA [15]. Stroke is the leading cause of long-term disability with only 12% of stroke survivors obtaining complete upper limb functional recovery.

The findings related to the use of RAT to facilitate upper extremity (UE) recovery in stroke rehabilitation are equivocal despite expected potential of RAT to maximize repetitive, task-specific training. Some studies have found significantly greater functional effects of RAT when compared to conventional therapy alone while many others have not. For instance, a systematic review and meta-analysis of 11 randomized controlled trials revealed that the effects of RAT and conventional therapy were similar, and that RAT combined with CT was not superior to CT alone [16]. Another study conducted at the University of Italy also reported similar findings [17]. However, Daly et al. reported that patients with stroke who underwent robotics and motor learning interventions made significant gains in the outcome measures related to arm function [18].

As research continues to evolve, it is essential to periodically review the effectiveness of novel interventions like RAT to determine their utility in practice. Further, despite mixed findings in terms of treatment effectiveness, RAT may be superior in other measures of recovery such as cost-benefit ratio and speed of recovery. This systematic review is an attempt to critically appraise the recent literature related to RAT in stroke upper extremity rehabilitation, with a specific focus on speed of recovery as it benefits all stakeholders including patients, service providers, and insurers. For the patients, faster recovery of function means a quicker return to optimal occupational performance. For the hospitals, a faster rate of recovery means more open beds and better patient outcomes [19]. For the insurers, a rapid recovery means less money spent on coverage of services. Speed in recovery is especially important when related to stroke rehabilitation due to the nature of neuroplasticity. The greatest improvement in function following a stroke tends to occur in the first six months which is an important window to target for greatest potential of recovered function [20]. Given the importance of this treatment window for stroke rehabilitation, it is critical to utilize interventions that are effective and help maximize functional recovery as quickly as possible within this time frame.

The authors believe that this research may yield insights related to the use of RAT in UE rehabilitation poststroke and the focus on speed of recovery may help rehabilitation professionals determine the logistics involved in stroke rehabilitation. No research was found to investigate speed of recovery using RAT, making the current study novel. Thus, this systematic review is aimed at investigating the following question: Is RAT effective in accelerating the UE functional recovery of patients with stroke when combined with conventional therapies, as opposed to conventional therapy alone?

## 2. Methods

The formation of the methods section was guided by the preferred reporting items for systematic review and meta-analyses [21].

### 2.1. Literature Search

We conducted a systematic search of articles published between 2011 and May 2021 on Ovid, MEDLINE, CINAHL, PubMed, and Scopus databases on 05/27/2021. All searches ran except those on Scopus limited the search results to articles published between 2011 and 2021.  Table 1 presents an overview of the literature search. All the 113 articles selected after title screening were uploaded to Rayyan, a web app for systematic review [22]. Eight articles were found to be duplicates and were removed. The remaining 105 articles were reviewed against the eligibility criteria.

Each of the first five authors reviewed a different database independently and imported chosen articles to Rayyan. Once all articles were in Rayyan, inclusion and exclusion decisions were made. The five authors' decisions were blinded to each other until all decisions were made. All five authors then reviewed each included article, and the final inclusion decisions were made by consensus.

### 2.2. Eligibility Criteria

**Figure 1:**
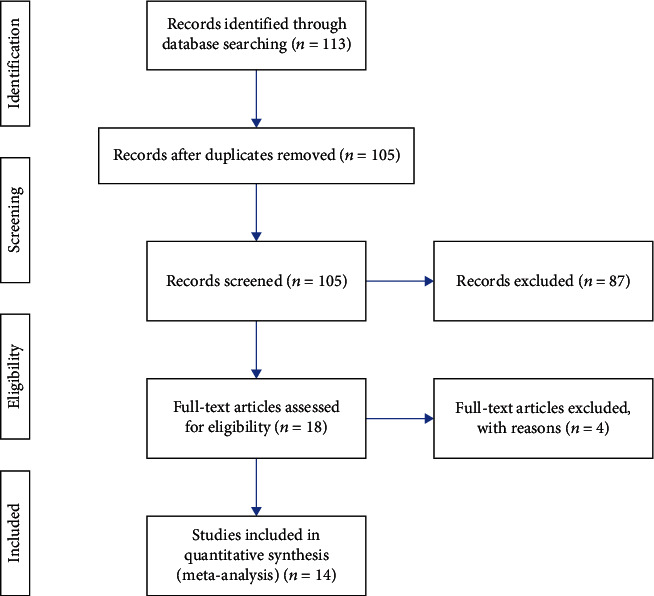
Search flow chart.

Articles were included only if the participants were adults (age > 18 years) with stroke resulting in functional deficits in their upper extremities. Articles were excluded if they enrolled children as participants or if the primary focus was on lower extremity function or gait. We considered articles that compared some form of RAT with any type of “conventional” therapies, such as motor relearning, functional electrical stimulation (FES), therapeutic exercise, proprioceptive neuromuscular facilitation, and constraint-induced movement therapy. Articles that were excluded did not compare RAT to conventional therapy. To be included, a trial had to use the Fugl-Meyer Assessment (FMA) as an outcome measure. We chose the FMA because it is widely known and was used by many of the studies reviewed. Many studies included a variety of outcome measures in addition to the FMA, but those that did not include the FMA were excluded to allow for standardized comparisons of recovery across included evidence. We excluded the articles published before 2011 to ensure recent evidence. Further, we selected only randomized controlled trials to include highest quality evidence. All articles with an evidence level lower than 2B, per the Center for Evidence-Based Medicine's (CEBM) Levels of Evidence table, were excluded [23]. Of the 105 articles reviewed, 87 were excluded during the screening process. The full texts of the remaining 18 articles were reviewed, and one more article was excluded because it had an evidence level lower than 2B. Three articles were later excluded because they did not include a control group (see Figure 1).

### 2.3. Data Extraction

The level of evidence of each article was determined using the American Occupational Therapy Association Guidelines for Systematic Review, which incorporates the CEBM evidence table [24]. These guidelines determine the level of evidence based on study design and quality. Five authors worked independently to extract the data from the articles. For each article, the level of evidence and risk of bias were determined. The following information was extracted from each article: number of participants, inclusion criteria, treatment setting, interventions that were used in the control and experimental group, outcome measures used, and results.

### 2.4. Risk of Bias

Risk of bias was assessed using the “Revised Tool for Assessing Risk of Bias in Randomized Trials” [25]. This tool evaluates each article based on the presence of random sequence generation, allocation concealment, baseline differences between groups, blinding of participants and study personnel, blinding of self-reported and objective outcome measures, incomplete outcome data, and selective reporting. Each study was identified as having low risk or high risk for each of these categories. The levels of risk were then summed to assess the overall risk of bias for each study. Not applicable (N/A) was selected for blinding of self-reported measures if the study did not utilize self-reported assessments. Each article was assessed for risk of bias by at least two authors. Disagreements were resolved through discussion and consensus.

### 2.5. Speed of Recovery

The primary outcome measure examined in this review was the speed of the UE functional recovery postrobotic therapy with concurrent CT. Speed of recovery was assessed by looking at the duration of the intervention and how much progress was made in that time, based on the FMA score. Using the formula of speed = distance (progress)/time, the change in FMA score was divided by total hours spent in the intervention to calculate progress made per hour.

## 3. Results

This systematic review included 14 randomized controlled trials. Most of the studies were from Taiwan and the US, and studies from China, Australia, Korea, Germany, Italy, Switzerland, and Japan were also included. Each selected study utilized a control and an intervention group. The control groups utilized a wide range of conventional stroke rehabilitative therapies, including, but not limited to, task training, passive range of motion, functional electrical stimulation, and modified constraint-induced movement therapy. The intervention group consisted of RAT which performed passive range of motion and helped support UE recovery along with some form of conventional therapy.

The number of total participants included in the 14 studies was 1,141 with a mean number of 41 participants per study. The number of participants in the studies ranged from 17 to 770. Participants were within the age range of 20 to 85 with a median age of 57 years.

Table 2 presents the summary of the evidence extracted. Twelve articles were rated as evidence level 1B, and the remaining two were rated as evidence level 2B. Four of the 14 studies had a moderate risk of bias while the remaining 10 had a low risk of bias. Due to the nature of the robotic therapy intervention, it was impossible to blind the participants to group assignment. Although some factors such as the inability to blind participants increased the risk of bias, most of the studies had moderate to low risk of bias because they utilized random assignment, did not have baseline differences between the intervention and control groups, and used blinding procedures during outcome assessments (see Table 3).

### 3.1. Speed of Recovery

Values for speed of recovery were calculated for both the robotic and control groups for comparison (see Table 4). The average speed of recovery of the robotic groups was calculated to be a 0.31 increase in FMA score per hour. Likewise, the average speed of recovery of the control groups was calculated to be a 0.24 increase in FMA score per hour. Because one study [35] only utilized the hand portion of the FMA, these values were calculated separately. The speed of recovery for this study was calculated to be 0.15 FMA points per hour for the robotics group and 0.04 FMA points per hour for the control group.

### 3.2. Intensity

**Figure 2:**
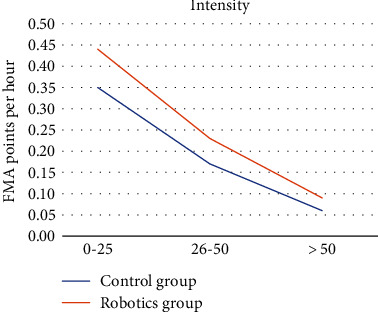
Intensity.

The change in FMA score was then divided by the time spent in the robotic intervention in each of the studies. The total intensity of each intervention was calculated in hours based on the length of each session, number of sessions per week, and number of weeks spent in intervention. The included studies were divided into three categories based on the number of hours that participants spent in the intervention: 10-25 hours, 26-50 hours, and more than 50 hours. Participants in seven studies spent 10-25 hours in intervention with an average of 15 hours [26–32]. Participants in three studies spent 26-50 hours with an average of 39 hours [33, 37, 39]. Participants in three other studies spent more than 50 hours with an average of 156 hours [34, 36, 38]. The change in FMA points per hour in the robotics group was compared to that of the control group for the three categories (see Figure 2).

The average speed of recovery demonstrated by the participants in the 10-25 hours group was calculated as 0.44 FMA points per hour, 26-50 hours group as of 0.23 FMA points per hour, and more than 50 hours group as 0.09 FMA points per hour.

Because intervention intensity differed drastically between studies, a daily intensity value was also calculated to show changes in FMA scores per hour per day. These values were calculated by dividing total hours spent in intervention by days spent in intervention (see Table 5).

**Figure 3:**
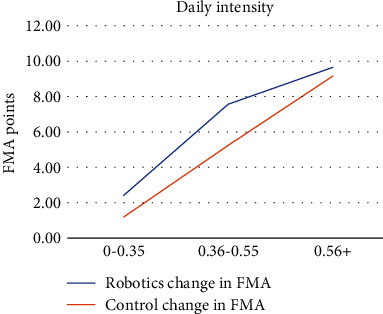
Daily intensity.

Studies were then grouped by daily intensity scores, and average change in FMA score was compared between robotics and control for each grouping as a measure of progress per hour per day: a daily intensity of low (0-0.35 hours/day), medium (0.36-0.55 hours/day), and high (0.56+ hours/day). Two studies used a low intervention intensity [31, 32]. Six studies used medium intensity [26–28, 30, 36, 39]. The remaining four studies used high intensity [29, 33, 37, 38]. The average changes in FMA robotics for low, medium, and high daily intensity studies were found to be 2.42 points/hour/day, 7.57 points/hour/day, and 9.65 points/hour/day, respectively. For the control groups, the average changes in FMA score for low, medium, and high daily intensity studies were found to be 1.19 points/hour/day, 5.24 points/hour/day, and 9.16 points/hour/day, respectively (see Figure 3).

### 3.3. Fugl-Meyer Scores

The difference between the baseline and end FMA_Total_ scores for the control group and the experimental group was calculated to determine the improvement in upper extremity function. Four of the included studies [26, 29, 34, 37] had a greater amount of improvement in the control group with none of them being a significant difference from the experimental, while nine studies [27, 28, 30–33, 36, 38, 39] had a greater improvement in the experimental group with three of them being significant. The study that only calculated the hand FMA scores showed greater improvement in the experimental group [35].

## 4. Discussion

This systematic review presents an overview of the current evidence on RAT in stroke rehabilitation with a special focus on the speed of recovery. It appears that the rate of improvement in UE functional recovery with RAT decreases after 25 hours spent in intervention. This may indicate that RAT may be effective only in the initial stages of the rehabilitation phase poststroke and that progress declines over time. Fading novelty is a phenomenon that demonstrates the waning of progress as an intervention becomes familiar [40, 41]. RAT may be an intervention that is subject to this phenomenon.

Further knowledge about this decline in the postintervention speed of progress would be beneficial to help determine the effect of RAT on the speed of stroke UE recovery over time. However, all included studies assessed only FMA scores at the start and the conclusion of the intervention period. The lack of intermediate FMA scores limited the author's ability to assess changes in speed over time. Nevertheless, significant functional progress was made within the first 25 hours in intervention which may support the use of RAT as a beneficial short-term intervention. Additionally, daily intensity scores showed greater improvement in UE functional recovery with both RAT and conventional therapy; however, RAT was shown to be slightly more effective. With both RAT and conventional therapy, greater time spent in therapy per hour per day results in greater functional improvement. This information proves that higher intensity of therapy following stroke is most beneficial for functional recovery, and RAT may help boost improvements made in the same amount of time.

### 4.1. Strength of Current Evidence

The findings show that RAT combined with conventional therapy did not significantly increase the speed of recovery when compared to conventional therapy alone over time. The evidence supporting this finding was rated as strong as this review appraised only high-quality evidence [42]. In addition, all reviewed articles had a low-to-moderate risk of bias.

### 4.2. Study Limitations and Future Directions

One limitation to this review was heterogeneity of variables. In addition, included articles examined stroke survivors of differing ages which could have impacted the recovery rate. Further, each study was performed with patients who were in different stages of stroke recovery ranging from six months to several years poststroke. This nonstandardized starting point poststroke also decreases clarity about whether functional progress was due to fading novelty or simply a normal plateau in progress. In addition, each of the studies reviewed used a different type of robot, which may also affect the rate of recovery.

An additional limitation is the differences in how RAT was used in each study. Intervention intensity among the studies included ranged from three days a week to seven days a week. Some studies had participants use RAT for 30 minutes a day while other studies used RAT for up to an hour. Finally, study duration ranged from 4 weeks to 12 weeks. The present study attempted to equalize some of these intensity differences by comparing intensity and speed using a variety of variables; however, the vast difference between included study designs is a limitation.

Future research could address these limitations by comparing studies with participants who are within the same age groups and the same length of time poststroke. Stratifying the articles into groups based on age and time since stroke could clarify the impact of these variables and help identify at which point in the recovery process the use of robotics would be the most beneficial. Future studies should also review articles that use more similar types of robots to increase the comparability of results and reduce extraneous factors that could impact the results. Reducing heterogeneity of the studies reviewed could better clarify the impact of robots as distinct from other factors such as age, time since stroke, and type of robot utilized.

In this review, a variety of conventional therapies were used such as repetitive task practice and constraint-induced movement therapy. Conventional therapies were not compared in the present review due to the vagueness of “usual care” used in some studies and the wide range of conventional therapies used. Future research could focus on comparing which of these conventional therapies is the most effective, then comparing that single CT to RAT. This method could help truly determine how RAT compares to CT.

### 4.3. Implications for Practice

The findings have the following implications for practice:

- Overall intensity of the interventions and functional progress had an inverse relationship; however, higher daily intensity demonstrated a positive correlation with functional progress. Hence, rehabilitation professionals may consider using higher-intensity RAT for a shorter duration in the beginning phases of rehabilitation
- Practitioners must exert caution and use their best professional judgment when choosing RAT as part of their intervention as several factors such as type of the device, stage of recovery, and cost and time associated with the intervention may influence the treatment outcome
- Practitioners must continue to appraise the recent evidence when making treatment decisions, especially when using novel interventions like RAT

## 5. Conclusion

The use of RAT in stroke rehabilitation appears to accelerate arm recovery in the first two weeks postintervention but needs more validation through empirical research. Current evidence regarding the use of RAT and its effect on the UE stroke recovery suffers from the variability in studies, technology used, implementation of RAT in different phases of recovery, etc. As speed of recovery has cost implications, practitioners must take that into consideration when developing stroke rehabilitation programs. More research examining the efficacy of RAT and speed of UE recovery poststroke is warranted.

## Figures and Tables

**Table 1:** An overview of the literature search.

Database	Search strings used	Number of hits received	Number of articles selected after title screening
Ovid	“exp Robotics” AND “motor impairment” AND [“exp Upper Extremity” AND “exp Stroke/or exp Ischemic Stroke/or exp Hemorrhagic Stroke/or exp Stroke Rehabilitation”]	55	4

CINAHL	(MH “Stroke+” OR MH “ischemic stroke+” OR MH “hemorrhagic stroke+”) AND (MH “robotics+” OR “exoskeleton devices”) AND (MH “Recovery+”) AND (MH “Upper Extremity+” OR MH “Shoulder” OR MH “Arm” OR MH “Hand”)	56	41
Scopus	Search 1: “Robot-assisted” AND “Stroke” AND “Function”	437	7
	Search 2: “Stroke” AND “Robotics”	5190	8

PubMed	Search 1: “stroke” AND “robotics”	950	17
	Search 2: “Upper limb” AND “stroke” AND “interventions” AND “occupational therapy”	2069	36

**Table 2:** Evidence table.

Author/year	Level of evidence Study design Risk of bias	Participants Inclusion criteria Study setting	Intervention and control groups	Outcome measures	Results
Aprile et al. [26]	Level 1B RCT Risk of bias: Moderate	Participants: *N* = 190 (56.8% men, 56.6% women, aged 40-85) Inclusion criteria: (i) Verified stroke (ischemic or hemorrhagic) (ii) Aged between 40 and 85 years old (iii) Time since the stroke is between 2 weeks and 6 months (iv) Cognitive and language abilities to understand intervention Intervention setting: multicenter	Intervention: Robotic group (*n* = 58) 25 sessions facilitated by a physical therapist. Session included treatment of proximal and distal UE with three different types of robotic devices. During treatment, subjects performed motor and cognitive tasks and received a vibratory treatment. Control group: Conventional therapy (*n* = 64) Daily therapy for 45 minutes, 5 days a week, for 30 sessions Subjects performed functional improvement through exercises, ADLs, sensory input, and meaningful activities to participants	Primary: FMA Secondary: MI, MRC, MAS, DN4, NRS, mBI, FAT, ARAT, Sf36, PCS, and MCS	There was no significant difference based on FMA between the intervention and control groups Predictors of recovery were identified as age and baseline FMA value

Carpinella et al. [27]	Level 2B Pilot RCT Risk of bias: Moderate	Participants: *N* = 38 adults poststroke (median age 67 y R_Group; 59 y C_Group, 47% female) Inclusion criteria: (i) First ischemic or hemorrhagic stroke (ii) A score between 1 and 3 on the upper limb subitem of the Italian version of the National Institutes of Health Stroke Scale (iii) A score higher than 6/66 on Fugl-Meyer Intervention setting: hospital	Intervention: Robot group (R_Group; *n* = 19) (i) Rehab treatment of affected upper limb consisting of 20 sessions, 45 min each, 5x/week (ii) Robot-based training using planar robotic manipulation for shoulder and elbow movements in the horizontal plane (iii) Robot had 2 modes: assist-as-needed and resistive Control: (C_Group; *n* = 19) (i) Rehab treatment of affected upper limb consisting of 20 sessions, 45 min each, 5x/week (ii) PROM, active mobilization of scapula, shoulder, elbow, wrist followed by task-oriented exercises	Instrumented assessments: (i) Shoulder/Elbow Coordination Index (ii) Measures of shoulder flexion, elbow extension, trunk compensation index Clinical assessments: (i) FMA (ii) Reaching Performance Scale (RPS) (iii) Modified Ashworth Scale (MAS) (iv) Functional Independence Measure (FIM)	Significant findings: Scores were significantly different between R_Group and C_Group on all instrumental assessments. R_Group showed larger improvement of interjoint coordination, elbow extension, and decrease in trunk compensation Nonsignificant findings: No significant difference between R_Group and C_Group scores on any of the clinical assessments

Hsu et al. [28]	Level 1B RCT Risk of bias: Low risk	Participants: *N* = 43 Inclusion criteria: (i) Diagnosis of stroke with unilateral cerebral infarction or hemorrhage whose time poststroke was more than six months (ii) No evidence of any other cerebral pathology (iii) Have an eligibility screening score on the Fugl-Meyer upper extremity motor assessment ranging from 23-53 corresponding with poor to notable arm-hand capacity (iv) No reported prestroke difficulties in verbal communication (v) No impairment was revealed in eligibility screening tests on the minimental state examination (MMSE) score above 24 and Lowenstein occupational therapy cognitive assessment (LOTCA), item scores at or above 8 for visual perception, 6 for spatial perceptions, 6 for praxis, and 14 for visuomotor organization (vi) Have prestroke right-handedness Intervention setting: community-dwelling setting	Intervention: (*n* = 22) Usual care which consisted of 10-minute sensorimotor stimulation session—repetitive upper limb range of motion exercises, proprioceptive neuromuscular facilitation using the Rood approach as well as the 40-minute robotic-assisted therapy with bilateral practice (RTBP) program for the wrist and forearm Control: (*n* = 21) Just “usual” care	Outcome measures: (i) Task performance using the Motor Activity Log (ii) Motor performance using sEMG (iii) FMA	Significant findings: (i) The RTBP (robotic therapy with bilateral practice) group, significant treatment effects have been found in the motor performance of total, wrist and shoulder parts as measured by the FMA Nonsignificant findings: (i) In the posthoc analysis, the hand component of the FMA-UE score significantly differed at the endpoint and follow-up only in the control group as compared to the baseline

Hesse et al. [29]	Level 1B Single-blind RCT Risk of bias: Moderate risk	Participants: *N* = 50 Inclusion criteria: (i) First-time supratentorial stroke (hemorrhagic or ischemic), lesion interval < eight weeks (ii) Age 18-90 years (iii) Able to get out of bed and mobilized in a wheelchair or were able to walk (iv) Participating in an inpatient rehabilitation program of at least six weeks (v) Nonfunctional or minimally functional upper limb (Fugl-Meyer score, (0 − 66) < 19, or Fugl-Meyer score 19-35) [15] (vi) No severe arm spasticity, i.e., scored 3 or less on the modified Ashworth Scale score (vii) No hemiparetic shoulder pain requiring physical therapy or pain medication (viii) No swollen hand impeding closing of the fist (ix) No other neurological or orthopedic arm impairments requiring physical therapy or pain medication (x) Able to give informed consent in the study Intervention setting: two inpatient neuro rehab centers	Intervention: (*n* = 25) (i) Patients received a 30-minute session of robot-assisted group therapy + a 30-minute individual arm therapy per workday for four weeks Control: (*n* = 25) (i) Patients received 2 × 30 min individual arm therapy sessions per workday for four weeks	Outcome: (i) FMA	Nonsignificant findings: (i) The blinded FMA score improvements during the intervention and follow-up period did not differ between groups (ii) The blinded FMA score improvements over time were significant in both groups Significant findings: (i) The blinded FMA score improvements over time were significant in both groups

Jiang et al. [30]	Level 1B RCT Risk of bias: Low	Participants: *N* = 45 (M age 64; 35% female) Inclusion criteria: (i) First ischemic or hemorrhagic stroke as confirmed by CT/MRI (ii) Age 35-85 years (iii) Less than 30 days since stroke (iv) Impaired upper limb motor function and unilateral hemiplegia (v) Sufficient cognition to understand purpose and instructions of study (vi) Ability to participate in robotic therapy (Brunnstrom 3-6) (vii) No visual problems Intervention setting: Inpatient ward of a hospital	Intervention: RAT (*n* = 23). Received therapy for 30 minutes twice a day, 5 days/week for 2 weeks. In addition, the RAT group received conventional rehabilitation therapy 30 minutes twice a day, 5 days/week for 2 weeks. Control: Conventional rehabilitation (CR) (*n* = 22). Received therapy for 30 minutes twice a day, 5 days/week for 2 weeks	Motor function: (i) FMA (ii) Motricity Index Activities of daily living: (i) Functional Independence Measure (ii) Barthel Index	Significant findings: There were positive effects on motor function and ADL after short-term RAT. Study shows that RAT can improve upper limb motor function in stroke patients as measured by FMA score

Klamroth-Marganska et al. [31]	Level 1B RCT Risk of bias: Low	Participants: *N* = 77 (27 females, 46 males) aged 18-80 Inclusion criteria: (i) Single CVA in a chronic state with moderate to severe arm paresis (ii) A difference of up to 3 points on the FMA-UE (iii) Minimum 18 years Intervention setting: Four clinical centers in Switzerland	Intervention: RAT (*n* = 39) Control: Conventional therapy (*n* = 38) Robotic and conventional therapy was applied for a period of 8 weeks, 3 times a week. The minimum time for therapy was 45 minutes	Primary: (i) Change in FMA-UE score Secondary: (i) WMFT (ii) The quality of 124 movement section of the Motor Activity Log (MAL-QOM) (iii) The Stroke Impact Scale (iv) The Goal Attainment scale (v) The Modified Ashworth Scale	Significant findings: Robotic training with ARMin reduced motor impairment with respect to arm and hand function more effectively than conventional therapy. About one third of the subjects in the ARMin group achieved meaningful gains (increase in FMA − UE ≥ 5 points), compared to one fourth in the control group Nonsignificant findings: Besides mean strength, no other secondary outcome measure showed significant differences in favor of either of the two treatments

Lee et al. [32]	Level 1B RCT Risk of bias: Moderate	Participants: *N* = 24 (64% male, 33.3% female, aged 21-74) Inclusion criteria: (i) First stroke with hemiplegia (ii) Subacute or chronic stroke (iii) Able to understand instructions (iv) Brunnstrom Stage II-V of recovery (v) Sensory impairments (vi) MAS score below 3 (vii) Aged 21-74 (viii) Able to see and hear feedback from device Intervention setting: Medical university hospital	Intervention: Robotic first group (*n* = 14) 12-hour sessions, facilitated by an occupational therapist. Session included the use of the Gloreha Sinfonia device, a glove that detects individual finger movements. During treatment, subjects performed weight bearing, rhythm activities, continuous whole-hand and individual finger PROM, and active assisted activities and games Control: conventional therapy first group (*n* = 10) 12-hour-long sessions Subjects performed weight bearing, rhythm activities, bilateral hand tasks, and pinch and grasp activities	Primary: FMA Secondary: Surface EMG, Semmes-Weinstein hand monofilament test, rNSA proprioception ranges, dynamometer, Box and Blocks test, MBI	Significant findings: The RAT first group showed significant improvement in UE motor control and ADL ability, and when compared to the conventional therapy first group showed more efficient hand extensor muscles

Lee et al. [33]	Level 1B RCT Risk of bias: Low	Participants: *N* = 30 (63.3% male, 36.6% female) Inclusion criteria: (i) Stroke induced hemiplegia within 6 months (ii) Able to communicate on their own (iii) 21 points on MMSE-K (iv) MAS below 2 (v) FMA score of minimally functional Intervention setting: Rehabilitation center	Intervention: Robotic group (*n* = 15) 40 30 min general OT sessions and 40 30 min RAT sessions, facilitated by an occupational therapist. Session included stretching exercises, neurodevelopmental therapy, resistance exercise, fine motor training, REJOYCE robot training with five functional activities Control: conventional therapy first group (*n* = 15) 40-hour-long sessions Session included stretching exercises, neurodevelopmental therapy, resistance exercise, and fine motor training	Primary: FMA Secondary: MBI	Significant findings: Both control and experimental groups showed significant increases posttherapy in FMA There was a statistically significant increase in all assessment scores when compared to the control group

McCabe et al. [34]	Level 1B RCT Risk of bias: Low	Participants: *N* = 39 Inclusion criteria: (i) Persistent (>1 y) upper extremity impairment (ii) Trace muscle contraction in wrist extensors (iii) Single unilateral stroke (iv) Mobility and function sufficient for independent performance of activities (v) Stable medical condition (vi) No other prior neurologic condition (vii) Ability to follow 2-step commands Intervention setting: Medical center	Intervention Group 1 (FES + ML; *n* = 12) Treatment 5 days/week for 5 hours/day for 60 sessions (1.5 h FES). Functional electrical stimulation used to stimulate finger flexors/extensors and forearm supinators/pronators Intervention Group 2 (ROB + ML; *n* = 12) Treatment 5 days/week for 5 hours/day for 60 sessions (1.5 h robotics). Robotics training using InMotion2Shoulder-Elbow Robot to target shoulder/elbow movements of flexion/extension and horizontal shoulder movements Control (ML; *n* = 11) Treatment 5 days/week for 5 hours/day for 60 sessions. Exercises provided for training-isolated joint movement coordination of scapula, shoulder, elbow, forearm, wrist, fingers, and thumb; task component movements; whole arm/hand functional training	Primary: (i) Arm Motor Ability Test (AMAT) Secondary: FMA	Significant findings: (i) All 3 groups showed significant improvement as measured by AMAT and FMA Nonsignificant findings: (i) No significant difference across group as measured by AMAT or FMA

Orihuela-Espina et al. [35]	Level 1B RCT Risk of bias: Low	Participants: *N* = 17 (M age 55 control, 56 intervention; 65% male) Inclusion criteria: (i) Adult patients (>30 years old) (ii) Diagnosis of hemorrhagic or ischemic stroke (iii) Experience severe upper extremity hemiparesis (Fugl-Meyer >8 and <30) Intervention setting: Neurologic rehabilitation unit	Intervention: RAT (*n* = 9) Treatment 5x/week for 40 sessions 1 hour each. Passive activities, partial assistance or resistance, active movements Control: Classical occupational therapy; (*n* = 8) Treatment 5x/week for 40 sessions 1 hour each. Treated with massage and conventional occupational exercises—passive motion, strengthening, fine motor	Primary: (i) FMA (ii) Motricity Index (MI)	Significant findings: (i) Improvement in FMA score for robotic intervention group and control group (ii) Improvement in FMA score was greater for the intervention group than for the control group

Rodgers et al. [3]	Level 1B RCT Risk of bias: Low	Participants: *N* = 770 (M age, 61 y; 61% male) Median time since stroke: 240 days Inclusion criteria: (i) Adults (ii) First stroke (iii) Stroke occurred between 1 week and 5 years ago (iv) Moderate to severe upper limb functional limitation (ARAT score 0-39) Intervention setting: four different outpatient centers in the UK	Intervention Group 1: RAT (*n* = 257) 45-minute sessions, 3 times per week for 12 weeks in addition to usual care Intervention Group 2: enhanced upper limb therapy (*n* = 259) 45-minute sessions, 3 times per week for 12 weeks in addition to usual care Control: Usual care (*n* = 254)	Primary: Action Reach Arm Test Secondary: (i) FMA (ii) Barthel ADL Index (iii) Stroke Impact Scale	Significant findings: (i) Enhanced upper limb therapy resulted in benefits in mobility, compared with usual care participants Nonsignificant findings: (i) Robot-assisted therapy did not result in significantly different stroke recovery

Sale et al. [36]	Level 1B RCT Risk of bias: Low	Participants: *N* = 53 (M age, 67 y; 22 female, 31 male) Inclusion criteria: (i) Subacute stroke patients (ii) First acute event of cerebrovascular stroke—unilateral paresis—ability to understand and follow simple instructions (iii) Ability to remain in a sitting posture Intervention setting: inpatient rehabilitation center	Intervention: The experimental group (robot-assisted proximal UE treatment) performed 30 sessions (5 days a week for 6 weeks) using the IM2 robot Control: The control group (usual physical therapy) received therapy 30 sessions (5 days a week for 6 weeks) of conventional rehabilitative treatment	Primary: (i) FMA (ii) The Modified Ashworth Scale Shoulder and Elbow Secondary: (i) PROM (ii) Motricity Index	Significant findings: (i) Intensive RAT in subacute stroke patients may significantly reduce motor impairment in the paretic upper limb Nonsignificant findings: n/a

Straudi et al. [37]	Level 1B RCT Risk of bias: Low	Participants: *N* = 40 (Median age, 68 y; 61.5% male) Median time since stroke: 37 days Inclusion criteria: (i) Diagnosis of 1st unilateral ischemic stroke verified by brain imaging (ii) Less than 8 weeks since stroke (iii) Upper limb impairment (score > 11 and <55 on the Fugl-Meyer) (iv) Age 18-80 (v) No other neurological conditions that may affect motor function Intervention setting: hospital	Intervention: 30 sessions (5 sessions/wk) of robot-assisted arm therapy and hand functional electrical stimulation (*n* = 20) Control: time-matched intensive conventional therapy (*n* = 20)	Primary: FMA Secondary: Wolf Motor Function Assessment, Modified Ashworth Scale, and the Barthel Index	Significant findings: Both groups significantly improved for all outcome measures Nonsignificant findings: There were no significant differences in recovery between the intervention group and the control group

Takahashi et al. [38]	Level 2B Prospective, exploratory randomized trial Risk of bias: Low	Participants: *N* = 60 Inclusion criteria: (i) Age between 20 and 80 years old (ii) First stroke in previous 4-8 weeks (iii) UE Brunnstrom stage II to IV movement Intervention setting: Inpatient stroke centers	Intervention: (*n* = 30) Received 40 minutes of standard therapy plus 40 minutes of RAT. Standard therapy consisted of UE exercises for stretching, range of motion, reaching, grasp, ADL training. RAT had 5 levels of assistance targeting proximal upper limb function Control: (*n* = 30) Received 40 minutes of standard therapy plus 40 minutes of self-guided therapy. Self-guided therapy techniques were selected from a list while a therapist supervised from a distance	Motor function: (i) FMA (ii) Wolf Motor Function Test (iii) Motor Activity Log	Significant findings: (i) Changes in FMA proximal UE and FMA flexor synergy were different between groups (ii) Lower UE function class (FMA < 30) had a greater increase in FMA score in the RAT group only Nonsignificant findings: (i) Change in total FMA UE score not different between groups

RCT: randomized controlled trial; UE: upper extremity; ADL: activities of daily living; FMA: Fugl-Meyer Assessment; ARAT: Action Reach Arm Test; PROM: passive range of motion; NMES: neuromuscular electrical stimulation; FES: functional electrical stimulation; TTT: transition to task therapy; WMFT: the Wolf Motor Function Test; SIS: Stroke Impact Scale; MI: Motricity Index; MRC: Medical Research Council; MAS: Modified Ashworth Scale; DN4: Neuropathic Pain Diagnostic Questionnaire; NRS: Neurological Rating Scale of Pain; mBI: Modified Barthel Index; FAT: Frenchay Arm Test; ARAT: Action Research Arm Test; SF36: 36-item Short Form Health Survey; PCS: Physical Composite Score; MCS: Mental Composite Score; rNSA: Revised Nottingham Sensation Assessment.

**Table 3:** Risk of bias.

Citation	Random sequence generation	Allocation concealment	Baseline differences b/w intervention groups	Blinding of participants	Blinding of study personnel	Blinding of outcome assessment—self-reported	Blinding of outcome assessment—objective	Incomplete outcome data	Selective reporting	Overall risk of bias
Aprile et al. [26]	+	—	+	—	—	+	+	—	—	Moderate
Carpinella et al. [27]	+	+	+	—	—	+	+	—	—	Moderate
Hsu et al. [28]	+	+	+	—	+	+	+	—	—	Low
Hesse et al. [29]	+	+	+	+	—	—	—	+	+	Moderate
Jiang et al. [30]	+	+	+	—	—	(n/a)	+	+	+	Low
Klamroth-Marganska et al. [31]	+	+	+	—	—	(n/a)	+	—	+	Low
Lee et al. [32]	+	+	+	—	—	—	—	+	—	Moderate
Lee et al. [33]	+	+	+	—	—	+	+	—	—	Low
McCabe et al. [34]	+	—	+	—	+	(n/a)	+	+	+	Low
Orihuela-Espina et al. [35]	+	—	+	—	—	(n/a)	+	+	+	Low
Rodgers et al. [39]	+	+	+	—	—	(n/a)	+	—	+	Low
Sale et al. [36]	+	+	+	—	+	—	+	+	—	Low
Straudi et al. [37]	+	+	+	+	—	(n/a)	+	—	+	Low
Takahashi et al. [38]	+	+	+	—	—	+	+	+	+	Low

*Note*. Key: yes (+), no (–), not sure (?), not applicable (n/a). Scoring for overall risk-of-bias assessment is as follows: 0–3 minuses, low risk of bias (L); 4–6 minuses, moderate risk of bias (M); 7–9 minuses, high risk of bias (H).

**Table 4:** Speed of recovery calculations.

Authors	Total hours	Total weeks	Change in FMA robotics (R)	Change in FMA control (C)	Speed of recovery R (FMA points/hour)	Speed of recovery C (FMA points/hour)
Aprile et al. [26]	22.50	6.00	8.50	8.57	0.38	0.38
Carpinella et al. [27]	15.00	4.00	7.00	6.20	0.47	0.41
Hesse et al. [29]	20.00	4.00	11.10	14.60	0.56	0.73
Hsu et al. [28]	10.00	4.00	4.50	2.20	0.45	0.22
Jiang et al. [30]	10.00	2.00	9.04	5.55	0.90	0.56
Klamroth-Marganska et al. [31]	18.00	8.00	3.25	2.47	0.18	0.14
Lee et al. [32]	12.00	6.00	1.58	-0.09	0.13	-0.01
Lee et al. [33]	40.00	8.00	8.20	2.33	0.21	0.06
McCabe et al. [34]	300.00	12.00	8.68	9.92	0.03	0.03
Orihuela-Espina et al. [35]	38.67	8.00	5.66	1.50	0.15	0.04
Rodgers et al. [39]	27.00	12.00	7.70	5.30	0.29	0.20
Sale et al. [36]	112.50	6.00	8.65	3.63	0.08	0.03
Straudi et al. [37]	50.00	6.00	9.80	12.80	0.20	0.26
Takahashi et al. [38]	56.00	6.00	9.50	6.90	0.17	0.12

**Table 5:** Daily intensity.

Authors	Daily intensity (hours/day)	Change in FMA robotics (R)	Change in FMA control (C)
Aprile et al. [26]	0.54	8.50	8.57
Carpinella et al. [27]	0.53	7.00	6.20
Hesse et al. [29]	0.71	11.10	14.60
Hsu et al. [28]	0.36	4.50	2.20
Jiang et al. [30]	0.36	9.04	5.55
Klamroth-Morganska et al. [31]	0.32	3.25	2.47
Lee et al. [32]	0.19	1.58	-0.09
Lee et al. [33]	0.71	8.20	2.33
McCabe et al. [34]	3.57	8.68	9.92
Orihuela-Espina et al. [35]		5.66	1.50
Rodgers et al. [39]	0.43	7.70	5.30
Sale et al. [36]	0.54	8.65	3.63
Straudi et al. [37]	0.71	9.80	12.80
Takahashi et al. [38]	0.67	9.50	6.90

## Data Availability

This is a systematic review. Data are the articles reviewed.

## Abbreviations

RAT: Robot-assisted therapy
CT: Conventional therapy
FMA: Fugl-Meyer assessment
UE: Upper extremity.
